# Intramuscular Transplantation of Muscle Precursor Cells over-expressing MMP-9 improves Transplantation Success

**DOI:** 10.1371/currents.RRN1275

**Published:** 2011-10-26

**Authors:** Christophe Pichavant, Cesare Gargioli, Jacques P. Tremblay

**Affiliations:** ^*^Department of Pharmacology, Emory University, Atlanta, Georgia, USA; ^†^Department of Biology University of RomeTor Vergata, Italy and ^‡^Professor, Department of Human Genetics, CHUL Research Center, Quebec City, Canada

## Abstract

Duchenne muscular dystrophy (DMD) is characterized by the absence of dystrophin in muscles. A therapeutic approach to restore dystrophin expression in DMD patient’s muscles is the transplantation of muscle precursor cells (MPCs). However, this transplantation is limited by the low MPC capacity to migrate beyond the injection trajectory. Matrix metalloproteases (MMPs) are key regulatory molecules in the remodeling of extracellular matrix (ECM) components. MPCs over-expressing MMP-9 were tested by zymography, migration and invasion assays in vitro and by transplantation in mouse muscle. In vitro, MPCs over-expressing MMP-9 have a better invasion capacity than control MPCs. When these cells are transplanted in mouse muscles, the transplantation success is increased by more than 50% and their dispersion is higher than normal cells. MMP-9 over-expression could thus be an approach to improve cell transplantation in DMD patients by increasing the dispersion capacity of transplanted cells.

## **Introduction**

Duchenne muscular dystrophy (DMD) is a genetic disease affecting 1 out of every 3500 boys [1]. This X-linked pathology is characterized by the absence of dystrophin in muscle fibers [2]. Dystrophin is linked to several proteins in a complex named dystrophin-associated glycoprotein complex (DGC) [3]. This complex is needed for the mechanical stability of muscle fibers and its absence weakens the sarcolemma and thus makes muscle fibers less resistant to physical and mechanical stress leading to a progressive muscle degeneration [4]. DMD patients die generally between 17 and 30 years of age. There are currently no curative treatments for this disease.

When a muscle is damaged, satellite cells surrounding the damage area are activated and migrate through the basement membrane to be incorporated into the wounded muscle fiber. They need to degrade the extracellular matrix (ECM) present between fibers to reach the damaged fibers [5]. Muscle precursor cells (MPCs) transplanted in muscle either fuse together to form new small fibers or fuse with existing host muscle fibers [6]. This forms hybrid muscle fibers containing endogenous host nuclei as well as nuclei of the transplanted donor MPCs [7]. It is thus possible to obtain dystrophin expression following the transplantation of non dystrophic MPCs into dystrophic muscles [8]. This has been done with success in dystrophic (*mdx*) mice [8][8]. The expression of a reporter gene present in the donor MPCs was also observed in the hybrid muscle fibers formed in non human primates [9]. Transplantation of normal MPCs in dystrophic muscles also restores the DGC expression [10]. Improvement of muscle fiber resistance is also observed in dystrophic muscles transplanted with normal MPCs [11] [12]. Moreover, some transplanted MPCs are able to generate functional donor-derived satellite cells allowing them to participate in further muscle regeneration [13] [14]. After the positive results obtained in *mdx* mice, clinical trials were rapidly undertaken in DMD patients (see ref [6] for an exhaustive list of these clinical trials).

However, some problems limit the success of this therapeutic approach such as the early cell death of transplanted MPCs and the immune response. This last problem can be resolved by immunosuppressing the DMD patients with tacrolimus [15]. Concerning the early cell death, between 70 to 80% of the grafted MPCs die 3-4 days after intramuscular transplantation [16] [17]. This rapid early cell death is not totally understood. Another problem limiting the success of this potential therapy is the poor migration/invasion capacity of the transplanted MPCs between or along the muscle fibers, which limits their fusion to only the fibers located near the injection trajectories.

When the MPC injection trajectories in non human primates and in DMD patients are at a 2 mm interval, clear rows of hybrid muscle fibers expressing a reporter gene or dystrophin are observed [15] [18]. However, such clear rows are not observed when the injection trajectories are 1 mm apart [18]. Indeed the transgene expression can reach up to 70% of the muscle fibers in this condition since there are more abundant muscle fiber damages, and thus more secreted chemokine factors and more ECM damages due to the numerous injection trajectories. Thus, MPCs need to degrade the ECM present between fibers to reach the damaged fibers [5]. The MPC capacity to migrate is thus an important factor to obtain a successful transplantation.

One way to increase the success of MPC transplantation is to pre-inject toxins, which increase the number of damaged muscle fibers and thus the number of fibers, which can be repaired [19]. Another way to increase the transplantation success is to irradiate the muscle before grafting MPCs [20]. This inhibits the proliferation of the recipient satellite cells, a condition that favors the participation of grafted cells in recipient muscle regeneration, and increases migration and proliferation of injected donor myoblasts in the long term [21][21]. However, these pre-treatments are not applicable in clinical trials. Another avenue to increase the MPC spreading is to enhance their capacity to degrade the ECM components. Matrix metalloproteases (MMPs) are key regulatory molecules in the degradation and remodeling of these components [22][22]. MMPs are indeed a family of enzymes that can selectively digest individual components of the ECM in many tissues. The over-expression of different MMPs to improve transplantation efficacy has previously been investigated (El Fahime on MMP-2 [23], Caron on MMP-7 [24], Wang on MMP-1 [25], and Morgan on MMP-2 and MMP-9 [26]). One of the MMPs, i.e., MMP-9, is able to degrade several ECM components such as collagens (type IV, V, XI and XIV), fibronectin and laminin (see ref [27] for an exhaustive list of its substrates). MMP-9 expression is increased following a muscle injury and it is known to be involved in muscle cell migration [5].

We have previously shown that the *in vivo* migration of tendon fibroblasts over-expressing MMP-9 was increased after intramuscular injection [28]. Since fibroblasts are not able to regenerate by injured muscles themselves, we have over-expressed MMP-9 in myogenic cells. Myogenic cells over-expressing MMP-9 (a subclone of the C2C12 cell line over-expressed MMP-9) have been previously used by Morgan *et al.* [26]. When this subclone was transplanted in mouse muscles, its transplantation success was improved in comparison to another sub-clone not expressing a high level of MMP-9. However, the increased transplantation success might indeed be due to the spontaneous invasiveness of C2 cells [29] or to the higher capacity of this subclone to fuse *in vitro*. We have thus decided to use primary cultures of MPCs. Dog MPCs were transplanted in mouse muscles to be able to distinguish host dystrophin (mouse) from donor dystrophin (dog) and thus to analyze the success of transplantation. Immunodeficient (Rag^-/-^) mice were used to avoid the rejection of dog MPCs since it is a xeno-transplantation [30].

In this article, we show that MPCs over-expressing MMP-9 transplanted in mouse muscles, previously irradiated or injured, migrated further than control MPCs, and therefore improved the transplantation success.

## Materials and Methods

### Animals

Rag^-/-^ mice were purchased from Charles River (Willington, MA). All the experiments performed on these animals were approved by the Animal Protection Committee of Laval University.

### Cell culture

293T cells were cultured in DMEM-HG (Gibco, Burlington, Canada) supplemented with 10% heat-inactivated fetal bovine serum (FBS, Invitrogen, Carlsbad, CA) and 1% penicillin-streptomycin (Gibco). Dog MPCs were obtained from a muscle biopsy of a 3 month old male Beagle (Marshall BioResources, North Rose, NY). Briefly, the muscle was cut into small fragments and digested with collagenase (2 mg/ml) during 1 hour at 37°C. The cell suspension was plated in proliferation medium [MB-1 medium (Hyclone, Mississauga, Canada) supplemented with 15% heat-inactivated FBS (Invitrogen), 1% penicillin-streptomycin (Gibco) and 10 µg/L of basic fibroblast growth factor (bFGF, Feldan, Quebec City, Canada)]. A 2-hours pre-plating period was performed to discard most of the fibroblasts. To verify the enrichment of MPCs, 2.5×10^5^ cells were stained for desmin (D33, 1:100, Dako, Mississauga, Canada) and NKH-1 (1:100, BD Bioscience, Mississauga, Canada) by flow cytometry. Cells were more than 90% positive for both markers (data not shown). To obtain myotubes, MPCs were cultured in differentiation medium [DMEM (Gibco) supplemented with 2% FBS (Invitrogen)]. All the cells were maintained at 37°C under 5% CO_2_.

### Lentiviral plasmid construction

The lentiviral vector coding for MMP-9 was previously described [28]. Briefly, a full-length mouse MMP-9 was cloned by PCR and inserted into a pLenti6/V5-D-TOPO vector (Invitrogen) by topoisomerase reaction. The construct was verified through sequencing and named pLeMMP-9.

### Lentiviral vector production

Self-inactivating lentiviral vectors were produced as previously described [31]. Our control lentiviral vector is the native pLenti6/V5-D-TOPO vector and cells transduced by this virus are referred as mock transduced. Briefly, psPAX2 (packaging vector plasmid), pMD2G (envelope plasmid) and the vector plasmid were transfected in 293T cells using CaCl_2_ precipitation. The medium was removed 16 hours post-transfection and the supernatant was harvested at 12, 24 and 36 h. Average titers were around 10^6^ TU/ml. MPCs were transduced with the lentiviral vectors with a multiplicity of infection of 100 in the presence of 8 µg/ml of polybrene (Sigma, Oakville, Canada). The medium was removed after 12 hours and cells were selected during 3 days with blasticidin (Invitrogen) at 15 µg/ml, starting 48 hours after the transduction.

### Protein harvest

To assess the gelatinase activity of MMP-9, the conditioned medium was harvested. Cells were washed with HBSS (Gibco) and incubated for 24 hours in a serum-free medium to have no serum proteins. After the incubation, conditioned media were collected and concentrated using a Centricon YM-30 (Millipore, Etobicoke, Canada).

To perform western blots, proteins from cells (not from the conditioned medium) were extracted by a methanol-chloroform technique.

### Zymography

To assess the gelatinase activity, 15 µg of proteins from the concentrated conditioned medium were loaded on 9% polyacrylamide gel containing 1.5 mg/ml gelatin (Laboratoire Mat, Quebec City, Canada). The gel was subsequently washed twice with a buffer (50 mM Tris-HCl, 2.5% Triton X-100) for 30 min and the gelatinase activity was promoted by incubating the gel overnight at 37°C in the activation buffer (50 mM Tris-HCl, 150 mM NaCl, 10 mM CaCl_2_, pH 7.6). After staining with 0.5% Coomassie brilliant blue during 30 min, gelatinase activity was visualized by a clear band at specific molecular weights.

### Proliferation and fusion assays

To measure the proliferation rate of the cells, MPCs were plated at 5×10^4^ cells/well in 6-well plates in proliferation medium. Cell populations were counted after 48 and 96 h with a hemacytometer. To evaluate the MPC differentiation, MPCs were plated at 6×10^4^ cells/well in 6-well plates in proliferation medium for 48 hours. Afterwards, the proliferation medium was replaced by the differentiation medium and the fusion index was evaluated 48 and 96 h later. To do so, cells were stained with a mouse anti-MHC antibody MF20 (1:100, DSHB, Iowa City, IA) followed by incubation with a goat anti-mouse IgG Alexa 546-conjugated (1:300, Invitrogen). The nuclei were stained with 4′,6-diamidino-2-phenylindole (1:10 000). The efficiency of the fusion was assessed by counting the number of nuclei in the myotubes (more than 2 nuclei) as a percentage of the total number of nuclei, referred as the fusion index. This was made by counting approximately 400 nuclei per well. Media were changed every 48 hours in both assays. The tested cell populations were untransduced, mock transduced and MMP-9 transduced MPCs. Each condition was tested in triplicate in 2 independent experiments.

### In vitro migration and invasion assays

24-well transwell assay dishes (Corning Life Sciences, Lowell, MA) were used to examine the migration of MPCs. Briefly, this modified Boyden chamber is a well (lower compartment) containing an insert (upper compartment). The upper and lower compartments are separated by an 8 µm pore size polycarbonate membrane. The evaluation of the MPC invasion was performed with the Chemicon collagen-based Cell Invasion Assay Kit (Chemicon international, Temecula, CA). The invasion chamber is similar to the chamber used for the MPC migration except that the bottom of the insert is an 8 µm pore size polycarbonate membrane covered by a layer of collagen matrix. Prior to cell seeding, transwell permeable supports were pre-incubated for 2 hours, in a serum-free proliferation medium, to improve cell attachment and spreading. For the migration test, 100 µl of a serum-free proliferation medium containing 3×10^5^ cells/ml was added to the upper compartment, whereas the lower chamber was filled with 600 µl of proliferation medium. For the invasion assay, 300 µl of a serum-free proliferation medium suspension containing 7×10^5^ cells/ml was added to the upper compartment, whereas the lower chamber was filled with 500 µl of proliferation medium. Migration assays were performed at 37°C for 14 hours whereas invasion assays were performed for 72 hours. Following the incubation periods, the inserts were removed and washed three times in phosphate-buffered saline (PBS) buffer. Cells remaining on the upper surface of the insert (non-migrated cells) were removed gently using a cotton swab. Cells on the lower surface were stained for 15 min in a solution containing 0.4% crystal violet, 2% ethanol and 0.1% ammonium oxalate for the migration assay and stained with the solution provided by the supplier during 20 min for the invasion assay. After staining, inserts were rinsed three times by dipping into PBS and left to air dry. Cells that had migrated through the pores of the polycarbonate membrane were visualized using a stereomicroscope. Pictures were taken and analyzed using the Scion Image software, a computer software modified from the NIH Image software of the National Institutes of Health, USA and publically available (Scion Corporation, Frederick, MD). The tested cell populations were untransduced and MMP-9 transduced MPCs with n = 5 for each group of the migration assays and with n = 4 for each group of the invasion assays. Each assay was tested in 2 independent experiments.

### Cell transplantation

Cell transplantations were performed in the *Tibialis anterior* (TA) of Rag^-/-^ mice. Two general conditions were used: multi-sites injections and single site injections. In the first one, 5×10^5^ MPCs suspended in 8 µL of HBSS (Gibco) were transplanted using 10 injections throughout each TA. Two groups of animals were transplanted. Mice were either g-ray irradiated (12 Gy) at the leg level to inhibit the proliferation of the recipient satellite cells or injected 2 days before the graft with 1 µg of cardiotoxin (Sigma) into the TAs to damage the muscle fibers. For single MPC transplantation, only 1 injection of 5×10^4^ cells was performed in TAs and mice were not previously irradiated nor injected with cardiotoxin. The grafted TAs were harvested 5 weeks after transplantation and were snap frozen in liquid nitrogen. Serial transverse 12 µm cryostat sections were obtained throughout the muscle. The tested cell populations were untransduced and MMP-9 transduced MPCs with n = 4 for each group.

### Histological analysis

Immunohistochemistry to detect dog dystrophin was performed with a mouse anti-dystrophin antibody MANDYS104 (1:8, 2 hours, CIND, Oswestry, UK),which reacts with dog but not mouse dystrophin. Afterwards, sections were incubated with a biotinylated anti-mouse antibody (1:300, 45 min, Dako) and with Streptavidin-Cy3 (1:300, 45 min, Sigma). After that, the sections were mounted in PBS-glycerol (1:1) and observed under fluorescence using an Axiophot microscope (Zeiss, Oberkochen, Germany).

The success of transplantation was evaluated for each transplanted muscle on serial 10 µm cryostat sections. The success was quantified by averaging the three highest numbers of dystrophin positive fibers observed throughout the muscle on cryostat sections spaced by at least 240 μm.

### Western blot

To perform western blots, 20 µg of proteins were loaded per well. The mouse MMP-9 was detected with a rabbit anti-MMP-9 antibody (1:2000, overnight, Abcam, Cambridge, MA) followed by incubation with a HRP anti-rabbit antibody (1:300, 45 min, Dako).

### Statistical analyses

The significance of results was evaluated by an analysis of variance (ANOVA) using a Fisher’s test PLSD. A p value < 0.05 was considered as statistically significant.

## Results

### MMP-9 over-expression

Lentiviral vector coding for mouse MMP-9 and a blasticidin resistance gene, and mock-lentiviral vector (pLenti6/V5-D-TOPO) were produced and tested on dog MPCs. Transduced cells were selected with 3 days exposure to blasticidin. Roughly 95% of the transduced cells were resistant to this antibiotic, meaning that the transductions of our lentiviral vectors and thus the expression of our transgenes were very effective. Blasticidin-resistant cells were cultivated in a proliferation medium for 2 more days and proteins were harvested. A western blot with an antibody against MMP-9 was done to confirm the expression of the transgene in the transduced cells. As expected, MMP-9 transduced cells expressed pro and active forms of mouse MMP-9 whereas control cells did not express it (Fig. 1A). Since the antibody used is specific to mouse and not to dog MMP-9, the endogenous MMP-9 expression of dog MPCs was not detected. To confirm the secretion and activity of MMP-9 in transduced cells, a zymography analysis was performed. The gelatinase activity of MMP-9 was increased in MMP9 transduced cells compared to untransduced and mock transduced cells (Fig. 1B). As expected, the gelatinase activity of MMP-2 did not change in the tested cells. Therefore, cells transduced by our lentiviral vector coding for MMP-9 were able to over-express and secrete a functional MMP-9 protein.

**Figure:**
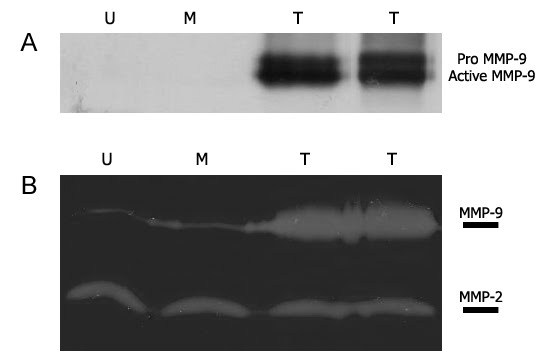
**Figure 1: MMP-9 over-expression. **(A) Western blot with 20 µg of protein. Two bands corresponding to MMP-9 products were observed in MMP-9 transduced MPCs. (B) Zymography with 15 µg of protein. MMP-9 activity was increased in MMP-9 transduced MPCs compared to control cells. MMP-2 activity was similar in each cell group. U: Untransduced cells, M: Mock transduced cells, T: MMP-9 transduced cells (cells coming from 2 different transductions were used). 

###  Proliferation and fusion assays

The effects of MMP-9 over-expression was tested on the proliferation and the differentiation of MPCs. Untransduced, mock transduced and MMP-9 transduced cells were plated at low confluence in the proliferation medium for 4 days. The MMP-9 over-expression did not enhance significantly the number of cells counted after 48 and 96 h (Fig. 2A). Untransduced, mock transduced and MMP-9 transduced cells were also plated at confluence in the differentiation medium for 2 and 4 days. The terminal differentiation was also evaluated by counting the number of nuclei in myotubes as a percentage of the total number of nuclei. No effects on the differentiation were observed between the cells tested at 48 and 96 h (Fig. 2B).

**Figure:**
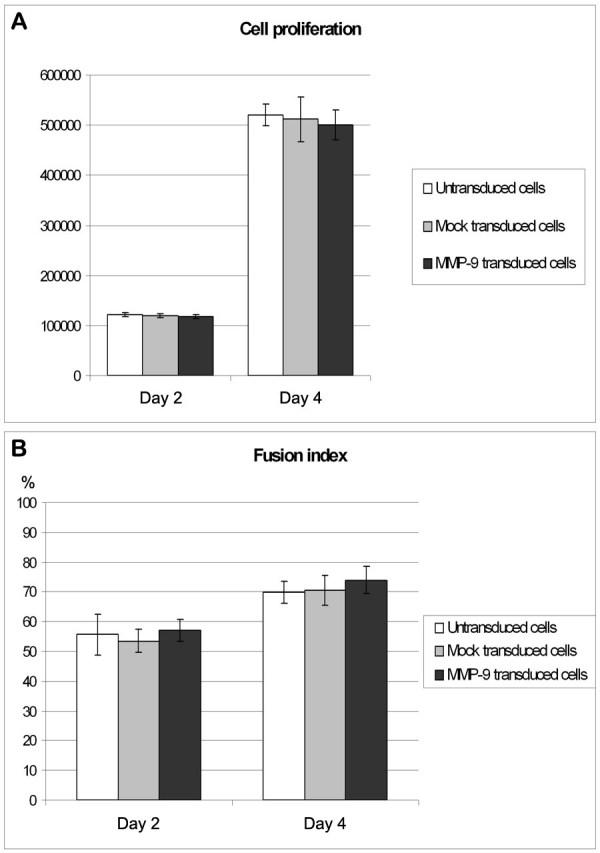
**Figure 2: Proliferation and fusion assays. **(A) Cell proliferation assay at 48 and 96 hours. No differences in proliferation were observed between untransduced, mock transduced and MMP-9 transduced cells. (B) Cell fusion assay at 48 and 96 hours. No differences in the fusion index were observed between untransduced, mock transduced and MMP-9 transduced cells. Two independent experiments were tested with n = 3 for each condition

### MMP-9 over-expression does not modify in vitro migration but increases in vitro invasion of MPCs

Since no differences in MMP-9 activity, proliferation and fusion were observed between untransduced and mock transduced cells, only untransduced and MMP-9 transduced cells were compared in the subsequent *in vitro* and *in vivo* experiments. A transwell assay was used to verify whether the MMP-9 over-expression had a chemokinetic property. Cells were placed in a serum-free medium located in the upper chamber of a transwell assay for 14 h. The membrane analysis showed that MMP-9 over-expression did not promote MPC migration (Fig. 3).

**Figure:**
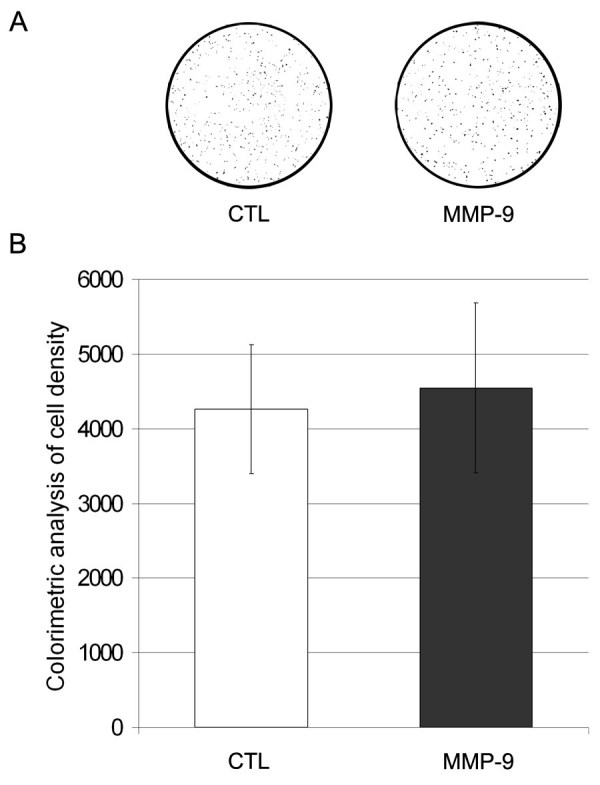
**Figure 3: Transwell migration assay.** 3×10^4^ cells in a serum-free proliferation medium were added to the upper compartment while the lower compartment contained proliferation medium. The upper and lower compartments were separated by an 8 µm pore size polycarbonate membrane. After a 14-hours incubation period, the inserts were removed, washed and colored. (A) The pictures shown are representative of the experiment and illustrate some of the analyzed pictures (where the backgrounds were removed). (B) Colorimetric analysis of cell density. For each picture, the area of points was measured using the Scion Image software giving the cell density. No significant differences were observed between the cells over-expressing MMP-9 and the untransduced cells. CTL: untransduced MPCs, MMP-9: MMP-9 transduced MPCs. Two independent experiments were tested with n = 5 for each group and for each experiment. 

 In order to verify whether the collagen of the basement membrane surrounding muscle fibers could be degraded by MPCs over-expressing MMP-9 *in vivo*, cells were placed in the upper chamber of an invasion assay for 72 h. Indeed, the* in vitro* invasion capacity of MMP-9 transduced cells was increased by more than 10-fold compared to untransduced cells (Fig. 4).

**Figure:**
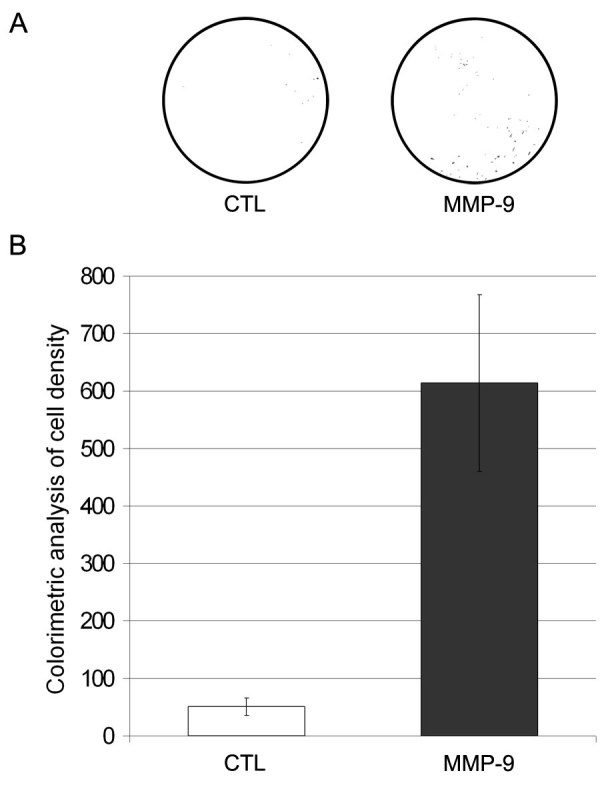
**Figure 4: Invasion assay.** 2.1×10^5^ cells in a serum-free proliferation medium were added to the upper compartment whereas the lower chamber was filled with proliferation medium. The bottom of the insert is an 8 µm pore size polycarbonate membrane covered by a layer of collagen matrix. Following a 72-hours incubation period, the inserts were removed, washed and colored. (A) The shown pictures are representative of the experiment and illustrate some of the analyzed pictures (where the backgrounds were removed). (B) Colorimetric analysis of cell density. For each picture, the area of points was measured using the Scion Image software giving the cell density. A significant difference was observed between untransduced and MMP-9 transduced MPCs with *p < 0.001. CTL: untransduced MPCs, MMP-9: MMP-9 transduced MPCs. Two independent experiments were tested with n = 4 for each group and for each experiment. 

###  MMP-9 over-expression in MPCs improves transplantation success

The invasion assay indicated that MPCs over-expressing MMP-9 were able to degrade ECM components better than control cells. To verify whether this was also the case *in vivo*, we have compared the transplantation success of control MPCs *vs* MPCs over-expressing MMP-9. The MPCs were transplanted in 2 different conditions, i.e., in mouse muscles previously irradiated or injected with cardiotoxin. Five weeks after the cell transplantation, muscles were harvested and the number of dog dystrophin-positive fibers was counted. In both conditions, the transplantation success of the MPCs over-expressing MMP-9 was increased by 65% and 72% respectively (Fig. 5).

**Figure:**
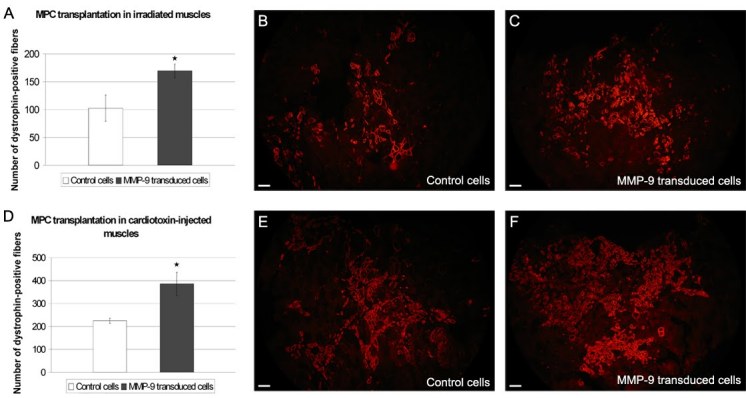
**Figure 5: MPC transplantation.** All the MPC transplantations were done in immunodeficient Rag^-/-^ mice. Ten perpendicularinjection trajectories were done in each muscle. (A,B,C) Cell transplantation in irradiated muscles. (D,E,F) Cell transplantation in muscles previously injected with cardiotoxin. (A,D) One month following cell transplantation, muscles were harvested and the graft success was evaluated by counting the number of dog dystrophin-positive fibers on each cryostat section. The number of dystrophin-positive fibers is the average of the 3 cross-sections having the highest number of dystrophin-positive fibers and separated by at least 240 µm. In both cases (in irradiated and cardiotoxin treated muscles), a significant difference was observed between the transplantation of untransduced and of MMP-9 transduced MPCs with *p < 0.01. (B,C,E,F) Fluorescent immunodetection of dog dystrophin (in red). (B,E) are representative cross-sections of the transplantation of untransduced MPCs and (C,F) are from muscles transplanted with MMP-9 transduced MPCs. Scale bar = 100 µm. The cross-sections represent the global result from the transplantation with n = 4 for each group

###  MMP-9 over-expression allows MPCs to migrate further

To evaluate the transplanted cell spreading, 5×10^4^ control MPCs and MPCs over-expressing MMP-9 were injected in Rag^-/-^ mouse muscles not irradiated and not injected with cardiotoxin. A single injection trajectory perpendicular to the muscle was done. Five weeks after the cell transplantation, muscles were harvested and analyzed. A graph representing the number of dog dystrophin-positive fibers for serial cryostat sections along the muscles was then made (Fig. 6A). Since these cryostat sections (12 µm-width) were spaced by 120 µm, the longitudinal migration of transplanted MPCs was evaluated by counting the number of cryostat section containing dog dystrophin-positive fibers for each transplanted muscle and multiplying this number by 120 µm. The longitudinal migration of the MPCs over-expressing MMP-9 was always superior to 1 mm and the average migration of this group was superior by 75% to that of the control cells (Fig. 6B). The total number of dog dystrophin-positive fibers counted in these cross-sections at 120 µm intervals was also increased when MPCs over-expressed MMP-9 (Fig. 6C).

**Figure:**
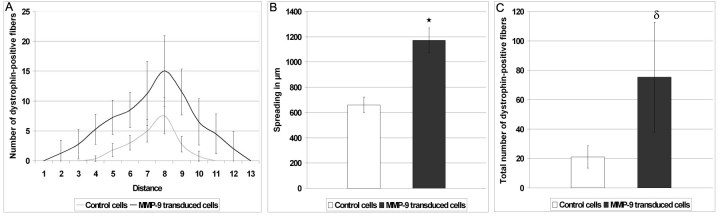
**Figure 6: Single perpendicular MPC transplantation per muscle.** (A) Representation of the number of dog dystrophin-positive fibers in function of the position along the TA muscle. Each position corresponds to a cryostat section. Each section is separated by 120 µm from the previous one. (B) Evaluation of the spreading of transplanted MPCs. The number of cryostat sections which contained at least 1 dystrophin-positive fiber was counted. A significant difference was observed between the spreading of untransduced and of MMP-9 transduced MPCs with *p < 0.001. (C) Total number of dystrophin-positive fibers counted on all cryostat sections. A significant difference was observed between the transplantation of untransduced and of MMP-9 transduced MPCs with dp < 0.05. n = 4 for each group. 

##  Discussion

The efficacy of MPC transplantation has been discussed in different animal models as well as in clinical trials on DMD patients. However, some problems limit the success of this therapeutic approach such as the poor migration/invasion capacity of MPCs. A simple way to increase transplantation success is to perform several injections with a high number of MPCs. Therefore, different factors were tested to enhance the poor migration of the grafted MPCs. IL-4 [32], insulin-like growth factor 1 (IGF-1) [33] [34], bFGF [34] [35] and mechanical growth factor (MGF) [36] have allowed to increase the *in vitro* MPC migration. Their *in vivo* migration, tested with the microtube technique, was also increased at short term [32] [34] [36] but did not improve the long term transplantation success except when MGF was injected weekly in the grafted muscles [37]. This can be explained by the fact that MPCs also need to degrade ECM surrounding muscle fibers to migrate further *in vivo* and thus to fuse with the damaged fibers. Another possibility to increase the *in vivo* MPC migration is to attract the MPCs towards the damaged muscle fibers. IL-13, released by damaged muscle fibers, would attract the MPCs towards these fibers. However, the potential improvement of MPC transplantation by this interleukin was not tested [38]. Moreover, even if the MPCs can respond to the chemoattactive signal produced by IL-13, they still need to be able to degrade the extra-cellular matrix. A recent study has also shown that MPCs migrate further in the muscle by extending the time in which they can proliferate after injection (e.g. by delaying differentiation) [39].

Our work was focused on the poor invasion capacity of MPCs. A lentiviral vector coding for MMP-9 was produced and tested on MPCs. The over-expression of MMP-9 showed no effects on MPC proliferation and fusion *in vitro*. This was expected since MMPs only have the capacity to degrade ECM even if a recent study has showed that another MMP (MMP-1) enhanced myoblast differentiation *in vitro* [25]. Nevertheless, this result needs to be confirmed with primary cell culture such as MPCs since this experiment was made with a C2C12 cell line. The *in vitro* migration of our MMP-9 transduced cells was not improved. However, their capacity to degrade ECM, in an* in vitro* invasion assay, was increased by more than 10-fold comparing to untransduced cells.

To confirm this result *in vivo*, intramuscular transplantations of MPCs over-expressing MMP-9 were done in mouse TAs. In mice previously g-ray irradiated or treated with cardiotoxin, the transplantation success of the MPCs over-expressing MMP-9 was increased by 65% and 72% respectively. While these two pre-treatments are very useful experimental tools to increase the success of transplantation, it is quite unphysiological and cannot be applied in clinical trials. In more physiological conditions (mice not irradiated nor injected with cardiotoxin) following single MPC transplantation, the longitudinal migration of the grafted MPCs over-expressing MMP-9 was improved by 75% reaching more than 1 mm. This shows that the ECM surrounding muscle fibers is normally a significant obstacle to the success of myoblast transplantation and that the degradation of this matrix by MMP-9 is one method to improve transplantation success without increasing the number of injection trajectory.

In our experiment, immunodeficient mice were used to avoid rejection issues due to the transplantation of dog MPCs in mouse muscles. In the context of DMD, our experiments need to be repeated under appropriate immunosuppression on dystrophic animals such as the *mdx* mouse and the dystrophic dog. In these animal muscles, it is known that there are more connective tissues and fibrosis, especially in the dystrophic dog. Is the MMP-9 over-expression sufficient to degrade this accumulation of ECM in dystrophic animals? This question is important to answer to consider the MMP-9 over-expression as one method to improve MPC transplantation success in DMD patients. Another point is that MMP-9 was over-expressed in MPCs by lentivirus transduction, and lentiviral vectors are known to randomly integrate in genome. Therefore, to be applicable in clinical trials, MMP-9 over-expression is MPCs needs to be induced by a safer method than lentivirus transduction. However, no drugs or chemical components are known to induce MMP-9 over-expression in myogenic cells.

## Acknowledgments

We thank Giulio Cossu for critical reading of the manuscript and helpful suggestions. We also thank Glenn E. Morris and Le Thanh Lam (MRIC Biochemistry Group, Wrexham, UK) for providing the MANDYS104 antibody.

## Funding information

This work was supported by a grant from the Association Française contre les Myopathies (AFM).

## Competing interests

Jacques P. Tremblay is the president and hold shares in CellGene inc., a biotech company involved in the development of cell and gene therapies.
